# Functional Signatures of Human CD4 and CD8 T Cell Responses to *Mycobacterium tuberculosis*

**DOI:** 10.3389/fimmu.2014.00180

**Published:** 2014-04-22

**Authors:** Teresa Prezzemolo, Giuliana Guggino, Marco Pio La Manna, Diana Di Liberto, Francesco Dieli, Nadia Caccamo

**Affiliations:** ^1^Dipartimento di Biopatologia e Biotecnologie Mediche e Forensi and Central Laboratory of Advanced Diagnosis and Biomedical Research, University of Palermo, Palermo, Italy

**Keywords:** *M. tuberculosis*, cytokines, human memory T cells, disease, infection

## Abstract

With 1.4 million deaths and 8.7 million new cases in 2011, tuberculosis (TB) remains a global health care problem and together with HIV and Malaria represents one of the three infectious diseases world-wide. Control of the global TB epidemic has been impaired by the lack of an effective vaccine, by the emergence of drug-resistant forms of *Mycobacterium tuberculosis* (Mtb) and by the lack of sensitive and rapid diagnostics. It is estimated, by epidemiological reports, that one third of the world’s population is latently infected with Mtb, but the majority of infected individuals develop long-lived protective immunity, which controls and contains Mtb in a T cell-dependent manner. Development of TB disease results from interactions among the environment, the host, and the pathogen, and known risk factors include HIV co-infection, immunodeficiency, diabetes mellitus, overcrowding, malnutrition, and general poverty; therefore, an effective T cell response determines whether the infection resolves or develops into clinically evident disease. Consequently, there is great interest in determining which T cells subsets mediate anti-mycobacterial immunity, delineating their effector functions. On the other hand, many aspects remain unsolved in understanding why some individuals are protected from Mtb infection while others go on to develop disease. Several studies have demonstrated that CD4^+^ T cells are involved in protection against Mtb, as supported by the evidence that CD4^+^ T cell depletion is responsible for Mtb reactivation in HIV-infected individuals. There are many subsets of CD4^+^ T cells, such as T-helper 1 (Th1), Th2, Th17, and regulatory T cells (Tregs), and all these subsets co-operate or interfere with each other to control infection; the dominant subset may differ between active and latent Mtb infection cases. Mtb-specific-CD4^+^ Th1 cell response is considered to have a protective role for the ability to produce cytokines such as IFN-γ or TNF-α that contribute to the recruitment and activation of innate immune cells, like monocytes and granulocytes. Thus, while other antigen (Ag)-specific T cells such as CD8^+^ T cells, natural killer (NK) cells, γδ T cells, and CD1-restricted T cells can also produce IFN-γ during Mtb infection, they cannot compensate for the lack of CD4^+^ T cells. The detection of Ag-specific cytokine production by intracellular cytokine staining (ICS) and the use of flow cytometry techniques are a common routine that supports the studies aimed at focusing the role of the immune system in infectious diseases. Flow cytometry permits to evaluate simultaneously the presence of different cytokines that can delineate different subsets of cells as having “multifunctional/polyfunctional” profile. It has been proposed that polyfunctional T cells, are associated with protective immunity toward Mtb, in particular it has been highlighted that the number of Mtb-specific T cells producing a combination of IFN-γ, IL-2, and/or TNF-α may be correlated with the mycobacterial load, while other studies have associated the presence of this particular functional profile as marker of TB disease activity. Although the role of CD8 T cells in TB is less clear than CD4 T cells, they are generally considered to contribute to optimal immunity and protection. CD8 T cells possess a number of anti-microbial effector mechanisms that are less prominent or absent in CD4 Th1 and Th17 T cells. The interest in studying CD8 T cells that are either MHC-class Ia or MHC-class Ib-restricted, has gained more attention. These studies include the role of HLA-E-restricted cells, lung mucosal-associated invariant T-cells (MAIT), and CD1-restricted cells. Nevertheless, the knowledge about the role of CD8^+^ T cells in Mtb infection is relatively new and recent studies have delineated that CD8 T cells, which display a functional profile termed “multifunctional,” can be a better marker of protection in TB than CD4^+^ T cells. Their effector mechanisms could contribute to control Mtb infection, as upon activation, CD8 T cells release cytokines or cytotoxic molecules, which cause apoptosis of target cells. Taken together, the balance of the immune response in the control of infection and possibly bacterial eradication is important in understanding whether the host immune response will be appropriate in contrasting the infection or not, and, consequently, the inability of the immune response, will determine the dissemination and the transmission of bacilli to new subjects. In conclusion, the recent highlights on the role of different functional signatures of T cell subsets in the immune response toward Mtb infection will be discerned in this review, in order to summarize what is known about the immune response in human TB. In particular, we will discuss the role of CD4 and CD8 T cells in contrasting the advance of the intracellular pathogen in already infected people or the progression to active disease in subjects with latent infection. All the information will be aimed at increasing the knowledge of this complex disease in order to improve diagnosis, prognosis, drug treatment, and vaccination.

## Introduction

Tuberculosis, with approximately 9 million cases annually, determines a world-wide mortality and morbidity, especially in low-income countries (1–3). *Mycobacterium tuberculosis* (Mtb), the causative agent of TB, is transmitted via aerosol droplets that are suspended in the air for prolonged periods of time (4), determining a risk of infection to people who inhalate these droplets. However, infection does not necessarily lead to TB disease; in fact, as reported in several studies, only 3–10% of immunocompetent individuals that are infected will develop the disease during their life-time (5), while more than 90% of infected subjects contain infection in a subclinical stage known as latent TB infection (LTBI), in which the pathogen remains in a quiescent state (4). One of the important aspects that can contribute to reactivation depends on the immune system of each individual that can be perturbed by several factors during life-time, such as chronic diseases: diabetes, alcoholic liver disease, HIV co-infection, and in some circumstances, the use of steroids or other immunosuppressive drugs. Another occurrence of active disease in later life is attributable to reactivation of latent Mtb bacilli or to a new infection with another Mtb strain. However, this huge reservoir contributes to fuel the high numbers of new active TB disease (3, 6); therefore, in order to diminish the risk of new active TB disease, it is important to treat LTBI cases by chemoprophylaxis, successfully eradicating the infection in the majority of cases. LTBI subjects, due to the increasing use of biological drugs, such as tumor necrosis factor-α (TNF-α)/Interleukin (IL)-12/IL-23 blockers for the treatment of inflammatory diseases like rheumatoid arthritis, Crohn’s disease, and psoriasis, have major risk to progress toward active disease more than other subjects (3, 7). Diagnosis of LTBI remains a priority for TB control within high income, low TB prevalence countries (8, 9), where a high proportion of TB cases occurs in immigrants from countries with high TB incidence (10, 11).

The study of subjects that are able to control Mtb infection in the long-term may be particularly informative in this respect. Despite two decades of intensified research, the mechanisms involved in the protective immune response against Mtb are not well understood. So, the comprehension of the pathways involved in protection in the host could represent biomarkers useful as correlates of protection, while the inhibition of the pathways involved in the surviving of host pathogens, could represent a biological target to contrast the bacilli growth and replication (12, 13).

*Mycobacterium tuberculosis* involves several conventional and unconventional T cell subsets that are characterized by distinct effector functions and surface phenotype markers (14). Th1 CD4 T cells activate effector functions in macrophages that control intracellular Mtb, and their role has been correlated with protection (14). Moreover, several studies have reported that Th17 cells, which are able to produce IL-17, are involved in immune protection against Mtb, primarily due to the effect of this cytokine in attracting and activating neutrophils (14, 15). Th17 cells have been involved in protection against TB at early stages (15, 16), for their capacity to recruit monocytes and Th1 lymphocytes to the site of granuloma formation (14, 15, 17). On the contrary, several studies have demonstrated that unrestricted Th17 stimulation determines an exaggerated inflammation mediated by neutrophils and inflammatory monocytes that rush to the site of disease causing tissue damage (14, 18–20).

CD4 T cells recognize antigenic peptides derived from the phagosomal compartment in the context of MHC-class II molecules (21). Mtb preferentially resides in the phagosome, where mycobacterial Ags can be processed and assembled to MHC-class II molecules (14, 22, 23). Another conventional lymphocytes subset, CD8 T cells, contributes to immune protection against TB (24): upon specific Ag recognition, CD8 T cells differentiate into effector cells, which produce cytolytic molecules and cytokines that kill both host cells and the intracellular Mtb (14, 25).

CD8 T lymphocytes recognize antigenic peptides, which are generally loaded in the cytosolic compartment in the context of MHC-class I molecules (21). MHC-class I loading can occur because of the intracellular pathogen or Mtb proteins diversification from the phagosome to the cytosol (14, 26). Moreover, apoptotic vesicles coming from infected macrophages and dendritic cells (DCs) can be uptaken by DCs (27, 28), which, in turn, will process and shuttled peptides into the canonical MHC-class I presentation pathway, a process termed cross-presentation (29).

Other cells play a role in the control or in the suppression of immune responses during Mtb infection such as Th2 cells, which counter-regulate Th1 cells and likely impair protective immunity against TB (30, 31), and regulatory T (Treg) cells (32, 33), which also contribute to the down modulation of the immune response to the pathogen (14) and to TB reactivation (14, 32–34).

**Figure 1:**
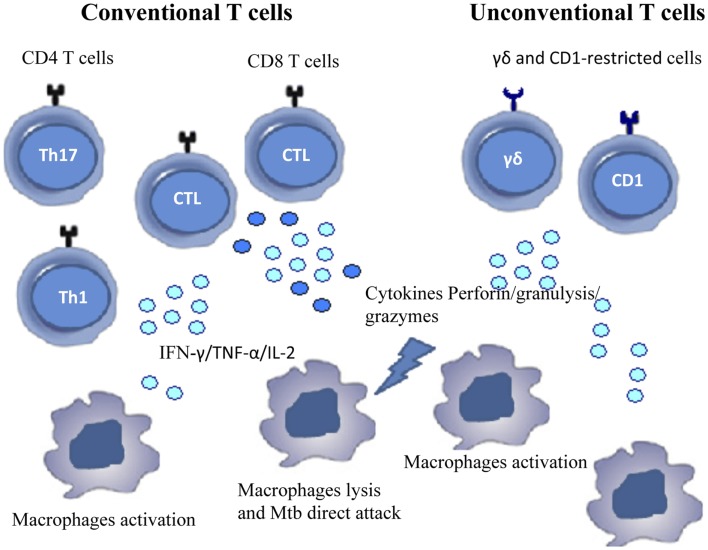
**Cells involved in immune response during Mtb infection**. The figure shows conventional and unconventional T cell subsets that contribute to the immune response against Mtb.

The so-called unconventional T cells are activated during TB; these cells are able to recognize lipids that are abundant in the mycobacterial cell wall, in the context of non-polymorphic CD1 molecules (35). Very recently, mucosal-associated invariant T cells (MAIT) have been found to recognize protein Mtb (Ags) presented by the non-classical molecule MR1 (36). γδ T cells, recognize “phosphoAgs” of host or bacterial origin and may also contribute to the immune response to Mtb as well (14, 37). Figure 1 shows the different cell populations involved in the immunopathology of TB.

In the last years, the potential role of distinct T cell subsets as biomarkers of active TB and/or LTBI has been studied. Functional CD4 and CD8 T cell subsets have been defined on the bases of cytokine production as single, double, or triple producer cells. These different cytokine signatures have been differently associated with disease stage, mycobacterial load or treatment, and several studies, mostly derived from vaccination in animals, have highlighted that polyfunctional CD4 T cells are associated with protective immunity. In contrast, more recent studies have suggested that these cells may be not correlated with protection, but rather with TB disease activity (38, 39).

In this review, we will analyze the complexity of the immune response of conventional CD4 and CD8 T cells widely described by recent studies in patients with pulmonary and extra-pulmonary disease and in subjects with LTBI, in order to better define the potential of different functional signatures of T cells as potential biomarkers.

## Populations of Human Memory T Cells

Individuals that have encountered a pathogen, develop an adaptive immune response with the induction of memory cells that will recognize the same Ag, upon the second encounter, dictating the type of immune response. Several studies have delineated that the quality of the memory response is important to dissect the real difference between protection and immunopathology, and to design strategies for vaccination (40).

Generally, the generation of memory T cells is characterized by different phases (41). The first encounter with an Ag, defined priming, determines a massive proliferation and clonal expansion of Ag-specific T cells followed by a phase of contraction, where the majority of these cells, named effector cells, are eliminated by apoptosis (42, 43). During this primary response, memory T cells develop and are maintained for extended periods due to several mechanisms such as the retention of Ag, stimulation/boosters, or homeostatic proliferation, that will insure the maintenance of a pool of cells that can rapidly respond to subsequent encounters with the pathogen.

The induction of memory T cells by vaccination against intracellular pathogens has definitively led a major challenge for the development of new subunit vaccines (40).

In humans, the functional properties of memory T and B cells can be defined, at least for those cells circulating in the blood, using techniques that detect typical surface markers (44). The combinatorial expression of surface markers such as adhesion molecules, chemokine receptors, and memory markers, allows for tissue specific homing of memory and effector lymphocytes and thus provides full characterization of that particular subsets of memory T cells, in terms of preferential residence inside tissues (40, 45, 46).

At least dozens of subsets can be identified and enumerated on the basis of distinct cellular functions that express unique combinations of surface and intracellular markers (47).

Memory T cells could be divided into CD62L^+^ and CD62L^−^ subsets; moreover some surface markers are specific for T cells homing to mucosa and skin that are confined to the CD62L^−^ subset (48, 49). The development of techniques that allow to measure cytokines production at the single-cell level and the analysis of several surface markers has permitted to correlate the functional properties of T cells with their phenotype (50). CCR7^+^ memory cells are named central memory (T_CM_) cells: they are able to home to secondary lymphoid tissues, produce high amounts of IL-2 but low levels of other effector cytokines (41), while their CCR7^−^ counter parts, named effector memory (T_EM_) cells, are able to produce high levels of cytokines, exert rapid effector functions and home to peripheral tissues (41). It has been established a relationship between T_CM_ and T_EM_ cells suggested by the analysis of the telomeres that are longer in T_CM_ than T_EM_ cells and T_CM_ cells are capable of generating T_EM_ cells *in vitro*, but not *vice versa* (41). Studies performed in humans and rhesus macaques both *in vitro* and *in vivo* have led to the identification of T cells with multiple stem cell-like properties, termed memory T stem cells (T_SCM_). These cells constitute a relatively rare memory population having a largely T naive (T_N_) phenotype, while overexpressing CD95 (51, 52), which is usually expressed at high levels by all memory cells (53, 54). T_SCM_ cells, precede T_CM_ cells in differentiation. These type of cells are capable of generating all memory subsets, including T_CM_ cells (51, 52); no other memory subset thus far has been found to regenerate T_SCM_ cells (44).

Another subset of “transitional” memory T cells (T_TM_) has been defined, mostly of which were isolated in the peripheral blood of healthy individuals (55, 56). These T_TM_ cells are more differentiated than T_CM_ cells but not as fully differentiated as T_EM_ cells in terms of phenotype (55, 56) and ability to expand in response to IL-15 *in vivo* (57, 58).

**Figure 2:**
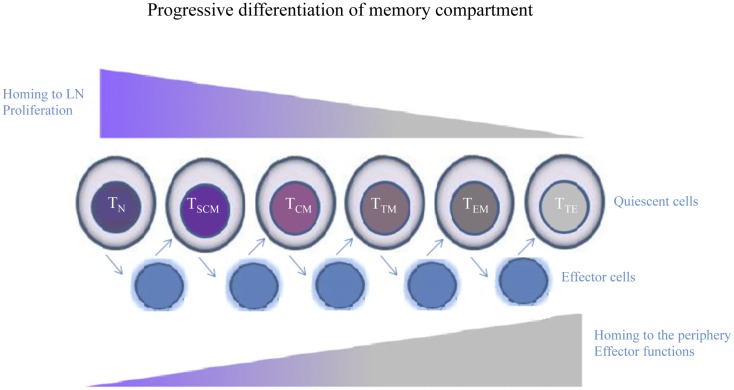
**Human memory T cell subsets**. Following encounter with Ag, quiescent T cells develop into effectors, whose phenotype is highly dynamic and largely unpredictable. When the Ag is cleared, effector T cells that survive return to a quiescent memory state. Cells differentiate from T_N_ to T_SCM_, T_CM_, T_TM_, T_EM_, and culminating in T_TE_ cells. Memory T cells progressively lose or acquire specific functions, such as the ability to migrate to peripheral tissues or to proliferate or produce effector molecules.

Very recently, Mahnke et al. propose that the phenotypic, functional, and gene expression properties of human memory T cell differentiation follow a linear progression along a continuum of major clusters (T_N_, T_SCM_, T_CM_, T_TM_, T_EM_, and T_TE_ cells) (44). According to this linear progression, memory T cells, progressively acquire or lose their specific functions (Figure 2). Other molecules that mediate lymphocyte functions, including markers of migration, co-stimulation, and cytotoxic molecules and adhesion markers can better define these different T cell subsets (Table 1).

**Table 1:** **Expression of functional molecules by circulating T cell subsets**.

Subsets	T_N_	T_SCM_	T_CM_	T_TM_	T_EM_	T_TE_	Category	Ag	Function
	+	++	++	++	−	−	Co-stimulation/survival	CD28	Co-stimulation
	++	+	+	+	±	−		CD27	Co-stimulation
	++	+++	+++	++	±	−		CD127	IL-7 signaling
	−	±	+	++	+	+		PD-1	Inhibition of effector function
	−	+	++	+++	+++	+++		CD122	IL-2/IL-15 signaling
	+	+	+	+	+	+		CD132	γc cytokine signaling
	−	ND	±	+	++	+++		KLRG-1	Inhibition of effector function
	+	++	++	+++	+++	+++	Adhesion	CD11a	Adhesion to APC/endothelium
	−	+	++	+++	+++	+++		CD58	Adhesion to APC
	±	+	++	++	++	++		CD99	Transendothelial migration
	+	+	+	−	−	−	Migration	CD62L	Secondary lymphoid tissues homing
	−	−	−	−	+	−		CD103	Gut homing
	±	+	++	+++	+++	±		CCR4	Chemokine response/Th2 associated
	−	−	+	++	+++	++		CCR5	Homing to inflamed tissues
	−	−	++	+++	+++	−		CCR6	Chemokine response/Th17 associated
CD4	−	ND	+	−	−	−		CCR9	Gut homing
CD8	−	ND	+	++	++	−			
	−	−	+	ND	++	−		CCR10	Skin homing
CD4	−	±	+	++	+++	+++		CXCR3	Homing to inflamed tissues
CD8	++	+++	+++	++	+	+			
	+	++	+++	+++	++	++		CXCR4	Homing to Bone Marrow
	−	ND	+	ND	++	ND		CLA	Skin homing
CD4	−	−	−	−	±	+	Cytolitic molecules	Granzyme A	Cleavage of cellular proteins
CD8	−	−	±	++	+++	+++			
CD4	−	−	−	−	±	±		Granzyme B	Cleavage of cellular proteins
CD8	−	−	−	+	++	+++			
CD4	−	−	−	−	±	±		Perforin	Pore forming
CD8	−	−	±	+	++	+++			

*Combination of + and – indicates the expression level respect to TN cells. ND = not determined*.

Seder et al. have proposed that T cells progressively acquire their functions with further differentiation, until they reach the phase that is adequate for their effector function (such as the production of cytokines or cytotoxic activity) (44, 59). The authors have demonstrated that the continued antigenic stimulation led to progressive loss of memory potential as well as the ability to produce cytokines, until the last step of the differentiation pathway represented by effector cells that are able to produce only IFN-γ and are short-lived, named terminally differentiated effector cells (T_EMRA_) (59). Another aspect that can optimize this linear differentiation process will depend on the amount of initial Ag exposure or the different conditions that are present in the microenvironment, which will dictate the extent of differentiation (44, 59).

Hierarchical expression of cytolytic molecules and surface markers, such as CD27, CD28, and CD57, has been delineated for CD8 T cell subsets. Granzyme (Gr)A is the first cytotoxic molecule detected in memory cells, followed by GrB and subsequently by perforin (60–62). GrB is always expressed in the presence of GrA, while, perforin^+^ cells are primarily positive for GrA and GrB, making it a choice indicator for cytolytic cells (62). Usually, perforin is present in cells that are CD27^−^ and CD28^−^ (63), while this molecule is always associated with the expression of the senescence marker CD57, which can be used as marker for T cells with high cytolytic potential (44, 62). Finally, the identification of the different subsets of human memory T cells, through the analysis of the expression of exclusive markers in that particular population could have a potential implications in T cell-based immunotherapy for infectious disease or other immune pathological conditions. Several studies have evaluated the different distribution of Ag-specific memory T cells subsets as good model of correlate of protection; for example, in response to chronic infectious agents such as HIV-1, hepatitis C virus (HCV), and Mtb, the increase of the frequency of Ag-specific T_CM_ cells, which produce high levels of IL-2, is associated with individuals’ ability to control the viral load (64–68).

Moreover, the response to cytokines used to differentiate or to maintain the different human memory T cells has been characterized (69). It has been shown that T_EM_ cells can proliferate in response to IL-7 and IL-15 *in vitro* but do not expand because of spontaneous apoptosis; conversely, T_CM_ proliferate and differentiate to T_EM_ cells, in the absence of these cytokines (70, 71).

Therefore, the quality of T cell responses can be modulated by several factors, and it is crucial for establishing the disease outcome in the context of various infections or pathologies.

In summary, the definition of the different subsets of memory T cells can be used to delineate the quality of a given T cell response, and this can be achieved by the combination of cell-surface phenotype, functional properties, and the capacity to traffic to lymphoid and non-lymphoid tissues: such a complex analysis should confer more intuition if an immune response will be protective or not.

## Subsets of Memory CD4 T Cells in TB

*Mycobacterium tuberculosis*-specific-CD4^+^ T cell protective response is typically due to Th1 cells and is mediated by IFN-γ and TNF-α that recruit monocytes and granulocytes and promote their anti-microbial activities (72–74).

Recent studies have shown that polyfunctional T cells (i.e., T cells equipped with multiple effector functions) (44, 75), could exert immune protection toward viral infections such as HIV (76, 77), models of TB vaccine (78–81), or in murine models of leishmania (36). However, the role of polyfunctional T cells during Mtb infection is controversial and different from that observed in chronic viral infections (36, 40, 81).

The definition of polyfunctional T cells was attributed to their ability to proliferate and to secrete multiple cytokines and these cells were found to play a protective role in antiviral immunity in chronic infections (when Ag load is low). Conversely, single IFN-γ-secreting CD4 and CD8 T cells typically predominate in acute infections (when Ag load is high), and in chronic infection characterized by the failure of immune control: in the case of HIV-1 infection, in fact, the response is dominated by HIV-1-specific-CD4 and -CD8 T cells that are able to produce only IFN-γ in both the primary and chronic phases of infection. On the other hand, the distinct cytokines profile during intracellular pathogens infection, comprises a very wide spectrum of T cell subpopulations (75).

Several authors have recently shown that polyfunctional T cells release multiple cytokines simultaneously in a relatively short period. The analysis of different aspects that could contribute to the release of cytokines, such as the methodologies used to stimulate the cells, peptides, or proteins used, the different cohort groups included in the study, should be taken into account, considering that very often the results obtained are controversial (75, 82).

Earlier studies in human TB have investigated on the role of polyfunctional T cells able to produce IFN-γ in combination with IL-2 (75, 83–86), and later on, a subset of cells able to simultaneously produce IFN-γ, TNF-α, and/or IL-2 was detected in patient with active TB disease compared to latently infected individuals (87–90), whose frequency decreased after anti-TB treatment. In another study, high frequencies of CD4 T cells expressing three cytokines simultaneously (IFN-γ, TNF-α, and IL-2) was found in adults with active TB disease, as compared to the frequency found in LTBI subjects, in which IFN-γ single and IFN-γ/IL-2 dual secreting CD4 T cells dominated the anti-mycobacterial response. Therefore, the presence of multifunctional CD4 T cells in TB patients was associated with the bacterial loads, as suggested by their decrease after completion of anti-TB chemotherapy (82, 91). This implies that multifunctional CD4 T cells are indicative of active TB rather than assuming a protective role. However, during these years, several contrasting findings have been reported, which do not allow a clear-cut conclusion on the role of polyfunctional CD4 T cells (40). In fact, some authors have found a reduced frequency of polyfunctional T cells in patients with active TB disease compared to latently infected individuals, which is recovered with the anti-TB therapy (75, 92, 93). Similar recovery of dual IFN-γ/IL-2-producing cells with the anti-TB therapy was also previously reported (82, 94).

Finally, a higher proportion of Ag-specific effector memory T_EM_ cells and a decreased frequency of T_CM_ CD4^+^ T cells has been found in patients with active TB (95, 96), as compared to the distribution found in LTBI individuals (75).

Since it is not possible to associate any specific cytokine profile with protection against active TB, recent studies have tried to find a correlation between functional signatures of CD4 or CD8 T cells and the state of infection/disease.

Marin et al. have analyzed the Th1 and Th17 responses through the counts of IFN-γ and IL-17 producing T cells by elispot assay, the frequencies of polyfunctional T cells producing IFN-γ, TNF-α, IL-2, and IL-17 by ICS, and the amounts of the above cited cytokines released after 1 day (short term) and 6 days (long-term) of *in vitro* stimulation using different Ags (CFP-10, PPD, or Mtb) (75) by ELISA. The evaluation of different T cell subsets after short- and long-term *in vitro* stimulation with different Ags has permitted to find a significant increase in single and double producer CD4^+^cells in long-term *in vitro* stimulation compared to short term *in vitro* stimulation in LTBI subjects and a significant increase of the frequency of single producer cells in patients with active disease (75). Mtb stimulation determined an increase in the frequency of single and triple producer T cells in LTBI subjects in 6 days compared to the frequency found in 1 day *in vitro* stimulated cells, with a significant value found for the frequency of double producer T cells in patients with active disease (75). These results suggest that the use of different mycobacterial Ags could induce distinct T cell functional signatures in LTBI subjects and in patients with active disease, highlighting that it is possible to define “functional signatures” of CD4 T cells correlated with the state of infection and that could be used as indicators of the clinical activity of the disease (82).

Very recently, Petruccioli et al. have correlated bifunctional “RD1-proteins”-specific-CD4 T cells with effector memory phenotype with active TB disease, while “RD1-proteins”-specific-CD4 T cells with a central memory phenotype were associated with cured TB and LTBI subjects (82). According to this study, the EM phenotype should be associated with inactive TB due to the presence of live and replicating bacteria, whereas the contraction of this phenotype and the further differentiation toward CM T cells in LTBI and cured TB subjects could indicate Mtb control, suggesting that the different expression of the memory/effector status may be used to monitor treatment efficacy, as previously suggested in patients with active TB with HIV co-infection (82, 97, 98).

A more detailed study on the role of Ag-specific T cell phenotype and function has been carried out by Lalvani et al. who delineated the association of TB disease stage with Mtb-specific cellular immunity. The authors have found the same trend of functional signature demonstrated by Petruccioli, but in response to different antigenic stimulation, namely PPD and RD1-peptides: in fact, Ag-specific-CD4 T cells were principally of the CM phenotype in subjects with latent infection compared to EM cells predominantly found in patients with active disease. Combined measurement of both functional profile and differentiation phenotype, in this study, reflects a discriminatory immunological status in the different cohort groups studied (patients with active disease vs. LTBI) (99). Moreover, HIV infection did not influence the number of Mtb-specific-CD4 effector cells, which instead was influenced by TB disease stage. This last aspect could be intriguing for the fact that assessment of cellular changes could be used also for immune compromised patients; in fact, it is known that HIV and active TB both impact Mtb-specific T cell immunity, such as skin test anergy, and therefore, dissection of distinct subsets as biomarkers could have an impact also in HIV co-infection.

Altogether, the above studies highlight the concept that the protective immune response against mycobacterial infection seems to depend more on the quality of CD4 T cell response assessed as the capacity to exert multiple functions, than on their magnitude, which is due to their Ag-specific frequency (44, 75). Finally, several methodologies used for the evaluation of the profiles of Mtb-specific-CD4 T cells in the reported studies led to different results: these include Ag specificity and type, *in vitro* stimulation conditions (short- or long-term *in vitro* stimulation), variability of the study cohort characteristics and at least, the monoclonal antibodies used to distinguish the subsets of CD4 T cells or intracellular cytokines content (40).

Thus, further studies are necessary to define particular phenotypes of Mtb-specific-CD4 T cells, assessing several functional properties such as activation, memory, migratory and inhibitory receptors, and ligands.

## Subsets of Memory CD8 T Cells in TB

CD8^+^ T cells contribute to protective response against TB (100, 101). CD8^+^T cells recognize Ags derived from an intracellular environment and could serve as sensors of bacterial burden. In fact, human CD8^+^T cells preferentially recognize cells heavily infected with Mtb (102) and in animal models, the magnitude of the CD8 response correlates with bacterial load (103–105).

The mechanisms involved in CD8^+^ T cell activation during Mtb infection are incompletely defined. DCs possess several pathways to load MHC-class I molecules, such as classical cytosolic processing, or alternative processing of phagosome located pathogens and endosome-located Ags. The recent evidences that virulent mycobacteria can escape from the phagosome into the cytoplasm and the possibility to direct access MHC-class I processing/presentation pathway provide a new mechanism (27). DCs also can take up vesicles derived from apoptotic Mtb-infected cells, after which the Ags are cross-presented through MHC-class I and class II molecules (28, 29). Finally, autophagy, which has a prominent role in cellular homeostasis and bacterial sequestration into vacuolar organelles, is involved in Ag presentation and cross-priming of T cells in response to intracellular pathogens, including Mtb (106, 107).

It has been demonstrated that several pathways are used in order to activate CD8^+^ T cells by phagosomal Ags, and, very recently, MHC-class Ib-restricted CD8^+^ T cells have received attention, including a role for HLA-E, which presents peptides from a wide range of mycobacterial Ags (34, 108). CD1-restricted CD8T cells recognize lipids such as mycolic acids and lipoarabinomannan from the bacterial cell wall (34) and lung MAIT recognize Mtb Ags in the context of the non-classical MR1 molecule (109).

Thus, CD8^+^ T cell immunity offers evidences of their clear synergy of action and complementarities in association with CD4^+^ T cell immunity, for the fact that CD8^+^ T cells display other direct effector functions such as the secretion of granules that contain cytotoxic molecules as perforin, granzymes, and granulysin. These molecules can lyse host cells, or can have a direct killing toward Mtb and other bacteria. Moreover, CD8^+^ T cells can induce apoptosis of infected target cells through molecules such as Fas or TNF-R family-related cell-death receptors. Finally, CD8^+^ T cells release, upon activation, cytokines such as IFN-γ, TNF-α, and in many cases also IL-2. These functions are also used by MHC-class Ib-restricted CD8^+^ T cells, suggesting a role for classical as well as non-classical CD8^+^ T cells in TB protection.

From the functional point of view, different studies conducted in mice and non-human models have delineated a role for Mtb-specific CD8^+^T cells in the control of Mtb infection (102–104). In these studies, it has been demonstrated that IFN-γ and perforin released by Mtb-specific CD8^+^ T cells were necessary to induce protection in Mtb-infected mice (102, 105). The role of these molecules has been efforted in humans’ studies that have reported the same conclusions (21, 110).

Hence, other *in vitro* studies have indicated that perforin- and/or granulysin-containing Mtb-specific CD8^+^ T cell lines were able to kill Mtb-infected macrophages or even free bacteria (25, 111, 112), other studies have found the complete absence of these molecules released by Mtb-specific CD8^+^ T cells from lung-associated tissues (113, 114).

Though it is not still possible to attribute a role to polyfunctional T cells as marker of protective immunity or of disease activity, multi-, or polyfunctionality of CD8 T cells is referred to the simultaneous production of several cytokines (IFN-γ, IL-2, TNF-α) and/or the expression of multiple effector functions (perforin, granulysin, cytolysis, etc.). However, contrary to initial expectations, these cells do not appear to correlate with BCG-induced protection in infants (115) and adults (116). Moreover, they are also present in active TB, although they may nevertheless be part of the protective host response attempting to limit infection rather than contributing to active disease.

Previously, we have correlated the frequency of Mtb-Ag85A-specific CD8^+^ T cells with the efficacy of anti-mycobacterial therapy in children. In particular, we found that Ag85A epitope-specific CD8^+^ T cells in children with active disease were able to produce low levels of IFN-γ and perforin, which recovered after successful therapy (117). In a later study, the analysis of the *ex vivo* frequencies, cytokine production, and memory phenotype of circulating CD8 T cells specific for different non-amers of Mtb proteins was performed in adult HLA-A*0201 different cohorts (87).

We found a lower percentage of circulating tetramer specific CD8 T cells in TB patients before therapy respect to LTBI subjects, but values increased after 4 months of anti-mycobacterial therapy to those found in subjects with LTBI. In this study, we also found high percentages of IL-2^+^/IFN-γ^+^ and single IFN-γ^+^ in subjects with LTBI, and a reduction of IL-2^+^/IFN-γ^+^ population in TB patients, suggesting a restricted functional profile of Mtb-specific CD8 T cells during active disease (87).

Many studies have focused on the response to different Mtb Ags expressed in the early phase of infection such as ESAT6, CFP-10, and Ag85B proteins but further studies should also incorporate those Ags expressed at different phases of infection (40).

Another study, using defined cohorts of individuals with smear-positive and smear-negative TB and LTBI subjects, evaluated Mtb-specific responses in correlation to mycobacterial load (93). The authors found, in individuals with high mycobacterial load smear-positive TB, a decrease of polyfunctional and IL-2-producing cells, and an increase of TNF-α^+^ Mtb-specific-CD4 T cells and CD8 T cells, both of which had an impaired proliferative capacity (40). These patients were followed during the anti-mycobacterial therapy and it was shown that the percentage of triple positive CD8 T cells (producing IFN-γ, IL-2, and TNF-α) increased over time in 7 out of 13 patients and this increase was paralleled by decrease of the frequency of IFN-γ^+^ T cells, providing another evidence that the cytokine production capacity of Mtb-specific CD8 T cells is associated with mycobacterial load.

In children or immunocompromised individuals, where it is very difficult to distinguish Mtb infection from disease, and in people that are at high risk to develop active disease, the increase of polyfuntional CD8 T cells and the reduction of single IFN-γ or TNF-α producing cells may be used to correlate these CD8 T cell subsets with TB disease progression, highlighting a new possible role as indicator of successful response to treatment.

*Mycobacterium tuberculosis* DosR-regulon encoded Ags (118) expressed by Mtb during *in vitro* conditions, represent rational targets for TB vaccination because they mimic intracellular infection. It has been shown that LTBI individuals are able to recognize Mtb DosR-regulon encoded Ags belonging to different ethnically and geographically distinct populations (40, 111, 118, 119). Moreover, Mtb DosR Ag-specific-CD4^+^ and -CD8^+^ polyfunctional T cells were found in LTBI subjects. In detail, a hierarchy of response, in terms of the ability of Ag-specific CD8 T cells to produce one or more cytokines, was found. The highest response was observed among single cytokine producing CD4^+^ and CD8^+^ T cell subsets, followed by double producing CD4^+^ and particularly CD8^+^ T cells. In particular, the most frequent multiple-cytokine producing T cells were IFN-γ^+^TNF-α^+^ CD8^+^ T cells. These cells were effector memory (CCR7^−^ and CD45RA^−^) or terminally differentiated effector memory (CCR7^−^ and CD45RA^+^) T cells, both phenotypes associated with the protective role of CD8^+^ T cells in Mtb infection (40, 111, 120). Another important observation was the number of epitopes identified, in accordance with their immunogenicity and recognition by a wide variety of HLA backgrounds (121, 122).

Therefore, the role of Mtb DosR-regulon encoded peptide Ag-specific single and double functional CD4^+^ and CD8^+^ T cell responses in LTBI, significantly improves the understanding of the immune response to Mtb phase-dependent Ags in the control of infection, and suggests a possible role for using MtbDosR-Ag and/or peptide based diagnostic tests or vaccination approaches to TB.

Several studies have tried to correlate the frequency, the phenotype, and the effector functions of CD8 T cells in patients with disease and subjects with latent infection. Here, we report other additional recent studies aimed at identify biological indicators useful to discriminate between patients with active disease, subjects with latent infection and patients that recovery after successful therapy.

Niendak et al. have observed that specific CD8^+^ T cell response decreased by 58.4% at 24 weeks, with the majority of the decrease (38.7%) noted at 8 weeks in subjects receiving successful anti-TB treatment (123); decrease of the CD8^+^ T cell response was relatively unaffected by malnutrition, supporting the hypothesis that the frequency of Mtb-specific CD8^+^ T cells declines with anti-tuberculosis therapy potentially as consequence of decreasing intracellular mycobacterial Ags, and may prove to be a surrogate marker of response to therapy (34, 124). The authors postulate that each individual has a CD8 “set point,” which reflects the complex interplay of antigenic exposure, in conjunction with host factors such as the HLA background. Nonetheless, these findings are concordant with the observation that removal of Ag results in decreasing T cell frequencies, and help to explain the observed reduction in CD8^+^ T cell frequency following anti-tuberculosis therapy.

Another recent study of Harari et al. (92) highlighted phenotypic and functional properties of Mtb-specific CD8 T cell responses in 326 TB patients and LTBI subjects in order to correlate their presence with different clinical form of Mtb infection (74). Authors found a higher frequency of Mtb-specific CD8 T cell responses in TB patients, which was correlated with the presence of higher Ag load (74, 92). These results were confirmed by two different studies, the first performed in children with active disease, where Mtb-specific CD8 T cells were detected in active TB disease but not in healthy children recently exposed to Mtb (92), and the second that demonstrated the presence of higher number of granulomas in TB patients as compared with those in LTBI subjects (74). Moreover, major phenotypic and functional differences were observed between TB and LTBI subjects, as Mtb-specific CD8^+^ T cells were mostly represented by terminally differentiated effector memory cells (T_EMRA_) in LTBI and of T_EM_ cells in TB patients. These results also suggests that T_EMRA_ and T_EM_ cell subsets, are involved in the control of Mtb infection, as already demonstrated in chronic controlled and uncontrolled virus infection, respectively (74, 125).

The authors did not find any statistically significant difference in the cytokines profile of Mtb-specific CD8^+^ T cell responses between LTBI subjects and TB patients, while they found that Mtb-specific CD8^+^ T cells were more polyfunctional (i.e., IFN-γ^+^TNF- α^+^IL-2^+^) in LTBI subjects, according to the role that these cells play in anti-viral immunity (74, 125). Instead, it was found that Mtb-specific CD8^+^ T cells have a higher frequency as single TNF-α-producer cells in TB patients, as occurred for CD4^+^ T cells (125). Further analysis of the functional properties of these Mtb-specific CD8^+^ T cells, permitted to detect significant high levels of GrB and GrA, but low level of perforin, suggesting a mechanism of action of Mtb-specific CD8^+^ T cells that is independent on the expression of perforin (74).

Another intriguing aspect of that study was the finding of a higher prevalence of Mtb-specific CD8^+^ T cell responses in pulmonary TB patients compared with extra-pulmonary TB patients and the higher magnitude of these responses in smear-positive versus smear-negative pulmonary TB patients (74). Moreover, Mtb-specific CD8^+^ T cells from pulmonary TB patients were not able to proliferate compared to CD8 T cells from extra-pulmonary TB patients (74). These functional differences of the CD8 T cell responses, in term of cytokines release or proliferation, most likely depend on antigenic stimulation that occur at different anatomic sites, that could be correlated with high Ag burden (88, 126, 127), attributing to tropism of responding T cells (74).

In conclusion, Mtb-specific CD8 T cell response, as defined by the qualitative and the quantitative aspects above cited, could have significance in understand how the immune system fails to control the progression of TB, or how the quality of the response could facilitate early diagnosis in order to reduce TB associated morbidity and mortality and to individuate subjects that are at high risk to develop active disease (40).

## Role of T Cells in TB-HIV Co-Infection

HIV infection has led to an increase in the incidence of TB, and TB-HIV co-infection has determined not easy decisions in both the diagnosis and treatment. The treatment of co-infected patients requires anti-tuberculosis and antiretroviral drugs to be administered together. The therapeutic treatment leads to different results, according to patient compliance, drug toxic effects, and, finally to a syndrome that appears following the initiation of antiretroviral therapy (ART) named immune reconstitution inflammatory syndrome (IRIS).

Several studies have provided to clarify the relationship that exists between HIV and Mtb pathogens and how they interact both *in vitro* and *in vivo*, highlighting how HIV infection could increase the risk of TB and how Mtb infection may accelerate the evolution of HIV infection. Flynn et al., very recently, have summarized the results obtained from different studies, discerning the several hypotheses on the role of the immune system in the co-infection (128).

It is well known that TB-HIV co-infection is destructive (129–131), but nowadays the mechanisms involved in the impairment of the immune system, guiding to the morbidity and mortality of co-infected subjects, remain to be elucidated (132). In countries with low rates of TB and, of course, with high-burden TB, the identification of LTBI within individuals co-infected with HIV is important due to the high risk to develop active TB. One of the control strategy adopted by the WHO is the use of preventive therapy of LTBI with isoniazid (INH) treatment (133). HIV-infected individuals are at high risk to develop active TB for the progressive CD4 depletion in the first few years after infection, even if the number of peripheral CD4 T cells is still high at the beginning (134–136). Although, the ART could restore absolute CD4 T cell numbers, it does not reduce the risk of TB progression in HIV patients (137). Conversely, TB infection has a negative impact on clinical progression of HIV infection (138).

Studies of human disease have characterized functional defects in CD4 T cells in TB-HIV co-infection by the analysis of cytokine production (e.g., IFN-γ) by CD4 cells in response to Mtb Ags (139–142) and by the analysis of phenotype distribution of CD4 T cells in lymphoid tissue, peripheral blood, and at the sites of disease (139, 143, 144). The correlation of different phenotypes of Ag-specific-CD4 T cells, and their role on the protection or susceptibility to infection, has been clearly demonstrated by the emerging characterization of polyfunctional CD4 T cells in TB-HIV co-infection. In the peripheral blood of TB-HIV-infected people, CD4 T cells are less able to secrete more than one cytokine when the viral load is high (145). Kalsdorf et al. have demonstrated that polyfunctional T cells specific for mycobacterial Ags are reduced in BAL from latent TB-HIV-infected subjects with no symptoms of active TB. The impairment of mycobacterial specific T cells could contribute to develop active TB, suggesting that HIV infection affects the frequency of Ag-specific polyfunctional T cells in the BAL of people with latent TB-HIV (140). Therefore, several studies have tried to correlate the presence of these cells in blood or in fluids recovered at the site of infection, highlighting how their presence can be reduced or increased, in term of absolute number. In fact, some authors have found a reduction of polyfunctional CD4 T cells in the peripheral blood of HIV-infected infants, in response to restimulation with BCG, compared with HIV-uninfected infants, or in BAL samples from HIV-infected subjects compared with HIV-uninfected healthy subjects, and finally, an increase in pericardial fluid of TB-HIV patients, with a terminally effector phenotype (143). Matthews et al. have found a lower proportions of Ag-specific polyfunctional T cells, with the less mature phenotype of CD4 T memory, at the site of disease of both HIV-infected and uninfected TB patients, supporting the hypothesis that their presence could correlate with Ag load and disease status, instead than with protection (143). Finally, understanding how the immune system contributes to TB-HIV co-infection could provide the basis for the discovery and development of new drugs and vaccines that can prevent or cure TB in co-infected people. At the moment, an early ART treatment still represents the gold standard in the control of TB-HIV co-infection.

## Concluding Remarks

Tuberculosis research in the field of vaccine and diagnostic tests development suffers from lack of rigorous correlates of protection in order to better understand the basic mechanisms underlying pathophysiology. Therefore, the identification of biosignatures that predict risk of disease, but also vaccine efficacy would be important.

Studies of human T cell responses, using different protocols of *in vitro* stimulation, have made possible to delineate some functional signatures indicative of the immunological status of each studied individual (40).

From the above cited studies, it has clearly emerged that, for TB diagnosis it is necessary to investigate on several biomarkers. The different expression levels of several cytokines, evaluated *ex vivo* in cells obtained from blood samples, comparing uninfected subjects, LTBI individuals, and patients with active disease, led to not unique results. This issue, therefore, requires further investigation by different analytical platforms. In particular, we believe that TB biomarkers research may continue to generate signatures with clinical applicability and additionally provides novel hypotheses related to disease pathophysiology (146).

Finally, the identification of such functional T cell signatures could help to better make diagnosis of different stages of TB, including also the cases of risk of reactivation and/or progression to active disease such as occurs in HIV patients (146).

## Conflict of Interest Statement

The authors declare that the research was conducted in the absence of any commercial or financial relationships that could be construed as a potential conflict of interest.

## References

[1] WHO. Global Tuberculosis Report 2011. Geneva: WHO Press (2011). Available from: http://whqlibdoc.who.int/publications/2011/9789241564380_eng.pdf

[2] Raviglione M Marais B Floyd K Lönnroth K Getahun H Migliori GB Scaling up interventions to achieve global tuberculosis control: progress and new developments. Lancet (2012) 379:1902–13 10.1016/S0140-6736(12)60727-2 22608339

[3] Whitworth HS Scott M Connell DW Dongés B Lalvani A IGRAs – the gateway to T cell based TB diagnosis. Methods (2013) 15:52–62 10.1016/j.ymeth.2012.12.012 23296020

[4] McNerney R Maeurer M Abubakar I Marais B McHugh TD Ford N Tuberculosis diagnostics and biomarkers: needs, challenges, recent advances, and opportunities. J Infect Dis (2012) 15:S147–58 10.1093/infdis/jir860 22496353

[5] Zumla A Atun R Maeurer M Mwaba P Ma Z O’Grady J Viewpoint: scientific dogmas, paradoxes and mysteries of latent *Mycobacterium tuberculosis* infection. Trop Med Int Health (2011) 16:79–83 10.1111/j.1365-3156.2010.02665.x 21342371

[6] Lillebaek T Andersen AB Dirksen A Smith E Skovgaard LT Kok-Jensen A Persistent high incidence of tuberculosis in immigrants in a low-incidence country. Emerg Infect Dis (2002) 8:679–84 10.3201/eid0807.010482 12095434 PMC2730343

[7] Keane J Gershon S Wise RP Mirabile-Levens E Kasznica J Schwieterman WD Tuberculosis associated with infliximab, a tumor necrosis factor alpha-neutralizing agent. N Engl J Med (2001) 345:1098–104 10.1056/NEJMoa011110 11596589

[8] National Collaborating Centre for Chronic Conditions (UK), Centre for Clinical Practice at NICE (UK). Tuberculosis: Clinical Diagnosis and Management of Tuberculosis, and Measures for Its Prevention and Control. London: National Institute for Health and Clinical Excellence (2011). Available from: http://www.nice.org.uk/nicemedia/live/13422/53642/53642.pdf 22720337

[9] Broekmans JF Migliori GB Rieder HL Lees J Ruutu P Loddenkemper R European framework for tuberculosis control and elimination in countries with a low incidence. Recommendations of the World Health Organization (WHO), International Union Against Tuberculosis and Lung Disease (IUATLD) and Royal Netherlands Tuberculosis Association (KNCV) Working Group. Eur Respir J (2002) 19:765–75 11999007 10.1183/09031936.02.00261402

[10] Pareek M Abubakar I White PJ Garnett GP Lalvani A Tuberculosis screening of migrants to low-burden nations: insights from evaluation of UK practice. Eur Respir J (2011) 37:1175–82 10.1183/09031936.00105810 21071474

[11] Abubakar I Lipman M Anderson C Davies P Zumla A Tuberculosis in the UK-time to regain control. BMJ (2011) 343:d4281 10.1136/bmj.d4281 21804155

[12] Parida SK Kaufmann SH The quest for biomarkers in tuberculosis. Drug Discov Today (2010) 15:148–57 10.1016/j.drudis.2009.10.005 19854295

[13] Mittrücker HW Steinhoff U Köhler A Krause M Lazar D Mex P Poor correlation between BCG vaccination-induced T cell responses and protection against tuberculosis. Proc Natl Acad Sci U S A (2007) 104:12434–9 10.1073/pnas.0703510104 17640915 PMC1941486

[14] Kaufmann SH Tuberculosis vaccines: time to think about the next generation. Semin Immunol (2013) 25:172–81 10.1016/j.smim.2013.04.006 23706597

[15] Perreau M Rozot V Welles HC Belluti-Enders F Vigano S Maillard M Lack of *Mycobacterium tuberculosis*-specific interleukin-17A-producing CD4+ T cells inactive disease. Eur J Immunol (2013) 43:939–48 10.1002/eji.201243090 23436562

[16] Ottenhoff TH New pathways of protective and pathological host defense to mycobacteria. Trends Microbiol (2012) 20:419–28 10.1016/j.tim.2012.06.002 22784857

[17] Jurado JO Pasquinelli V Alvarez IB Peña D Rovetta AI Tateosian NL IL-17 and IFN-γ expression in lymphocytes from patients with active tuberculosis correlates with the severity of the disease. J Leukoc Biol (2012) 91:991–1002 10.1189/jlb.1211619 22416258 PMC3360475

[18] Auffray C Sieweke MH Geissmann F Blood monocytes: development, heterogeneity, and relationship with dendritic cells. Annu Rev Immunol (2009) 27:669–92 10.1146/annurev.immunol.021908.132557 19132917

[19] Chowdhury D Lieberman J Death by a thousand cuts: granzyme pathways of programmed cell death. Annu Rev Immunol (2008) 26:389–420 10.1146/annurev.immunol.26.021607.090404 18304003 PMC2790083

[20] Nathan C Neutrophils and immunity: challenges and opportunities. Nat Rev Immunol (2006) 6:173–82 10.1038/nri1785 16498448

[21] Dorhoi A Reece ST Kaufmann SHE Fundamental immunology. 7th ed In: Paul WE editor. Immunity to Intracellular Bacteria. Philadelphia: Wolters KluwerHealth, Lippincott Williams & Wilkins (2012). p. 973–1000

[22] Harding CV Boom WH Regulation of antigen presentation by *Mycobacterium tuberculosis*: a role for Toll-like receptors. Nat Rev Microbiol (2010) 8:296–307 10.1038/nrmicro2321 20234378 PMC3037727

[23] Kaufmann SHE Fundamental immunology. 5th ed In: Paul WE editor. Immunityto Intracellular Bacteria. Philadelphia, NY: Lippincott-Raven (2003). p. 1229–61

[24] Cooper AM Cell-mediated immune responses in tuberculosis. Annu Rev Immunol (2009) 27:393–422 10.1146/annurev.immunol.021908.132703 19302046 PMC4298253

[25] Stenger S Hanson DA Teitelbaum R Dewan P Niazi KR Froelich CJ An antimicrobial activity of cytolytic T cells mediated by granulysin. Science (1998) 282:121–5 10.1126/science.282.5386.121 9756476

[26] Van der Wel N Hava D Houben D Fluitsma D van Zon M Pierson J *M. tuberculosis* and *M. leprae* translocate from the phagolysosome to the cytosol in myeloid cells. Cell (2007) 129:1287–98 10.1016/j.cell.2007.05.059 17604718

[27] Schaible UE Winau F Sieling PA Fischer K Collins HL Hagens K Apoptosis facilitates antigen presentation to T lymphocytes through MHC-I and CD1 in tuberculosis. Nat Med (2003) 9:1039–46 10.1038/nm906 12872166

[28] Winau F Weber S Sad S de Diego J Hoops SL Breiden B Apoptotic vesicles crossprime CD8 T cells and protect against tuberculosis. Immunity (2006) 24:105–17 10.1016/j.immuni.2005.12.001 16413927

[29] Lin MY Ottenhoff TH Host-pathogen interactions in latent *Mycobacterium tuberculosis* infection: identification of new targets for tuberculosis intervention. Endocr Metab Immune Disord Drug Targets (2008) 8:15–29 10.2174/187153008783928398 18393920

[30] Mosmann TR Coffman RL TH1 and TH2 cells: different patterns of lymphokine secretion lead to different functional properties. Annu Rev Immunol (1989) 7:145–73 10.1146/annurev.iy.07.040189.001045 2523712

[31] Romagnani S “Immunology. 10th ed In: Kaufmann SHE Steward MW editors. Cytokines. London: Hodder Arnold/ASM Press (2005). p. 273–99

[32] Keir ME Butte MJ Freeman GJ Sharpe AH PD-1 and its ligands in tolerance and immunity. Annu Rev Immunol (2008) 26:677–704 10.1146/annurev.immunol.26.021607.090331 18173375 PMC10637733

[33] De Libero G Mori L Recognition of lipid antigens by T cells. Nat Rev Immunol (2005) 5:485–96 10.1038/nri1631 15928680

[34] Gold MC Cerri S Smyk-Pearson S Cansler ME Vogt TM Delepine J Human mucosal associated invariant T cells detect bacterially infected cells. PLoS Biol (2010) 8:e1000407 10.1371/journal.pbio.1000407 20613858 PMC2893946

[35] Scotet E Nedellec S Devilder MC Allain S Bonneville M Bridging innate and adaptive immunity through gammadelta T-dendritic cell crosstalk. Front Biosci (2008) 13:6872–85 10.2741/3195 18508701

[36] Darrah PA Patel DT De Luca PM Lindsay RW Davey DF Flynn BJ Multifunctional TH1 cells define a correlate of vaccine-mediated protection against Leishmania major. Nat Med (2007) 13:843–50 10.1038/nm1592 17558415

[37] Derrick SC Yabe IM Yang A Morris SL Vaccine-induced anti-tuberculosis protective immunity in mice correlates with the magnitude and quality of multifunctional CD4 T cells. Vaccine (2011) 29:2902–9 10.1016/j.vaccine.2011.02.010 21338678

[38] Joosten SA Ottenhoff TH Human CD4 and CD8 regulatory T cells in infectious diseases and vaccination. Hum Immunol (2008) 69:760–70 10.1016/j.humimm.2008.07.017 18835413

[39] Urdahl KB Shafiani S Ernst JD Initiation and regulation of T-cell responses in tuberculosis. Mucosal Immunol (2011) 4:288–93 10.1038/mi.2011.10 21451503 PMC3206635

[40] Caccamo N Dieli F Are Polyfunctional Cells Protective in M. tuberculosis Infection? Understanding Tuberculosis – Analyzing the Origin of Mycobacterium tuberculosis Pathogenicity. Available from: http://www.intechopen.com/books/understanding-tuberculosis-analyzing-the-origin-of-mycobacterium-tuberculosis-pathogenicity/are-polyfunctional-cells-protective-in-m-tuberculosis-infection-

[41] Sallusto F Lenig D Förster R Lipp M Lanzavecchia A Two subsets of memory T lymphocytes with distinct homing potentials and effector functions. Nature (1999) 401:708–12 10.1038/44385 10537110

[42] Lanzavecchia A Sallusto F Understanding the generation and function of memory T cell subsets. Curr Opin Immunol (2005) 17:326–32 10.1016/j.coi.2005.04.010 15886125

[43] Zanetti M Franchini G T cell memory and protective immunity by vaccination: is more better? Trends Immunol (2006) 27:511–7 10.1016/j.it.2006.09.004 16997631

[44] Mahnke YD Brodie TM Sallusto F Roederer M Lugli E The who’s who of T-cell differentiation: human memory T-cell subsets. Eur J Immunol (2013) 43:2797–809 10.1002/eji.201343751 24258910

[45] Butcher EC Picker LJ Lymphocyte homing and homeostasis. Science (1996) 272:60–6 10.1126/science.272.5258.60 8600538

[46] Sallusto F Mackay CR Lanzavecchia A The role of chemokine receptors in primary, effector, and memory immune responses. Annu Rev Immunol (2000) 18:593–620 10.1146/annurev.immunol.18.1.593 10837070

[47] Appay V van Lier RA Sallusto F Roederer M Phenotype and function of human T lymphocyte subsets: consensus and issues. Cytometry A (2008) 73:975–83 10.1002/cyto.a.20643 18785267

[48] Picker LJ Treer JR Ferguson-Darnell B Collins PA Buck D Terstappen LW Control of lymphocyte recirculation in man. I. Differential regulation of the peripheral lymph node homing receptor L-selectin on T cells during the virgin to memory cell transition. J Immunol (1993) 150:1105–21 7678616

[49] Picker LJ Treer JR Ferguson-Darnell B Collins PA Bergstresser PR Terstappen LW Control of lymphocyte recirculation in man. II. Differential regulation of the cutaneous lymphocyte-associated antigen, a tissue-selective homing receptor for skin-homing T cells. J Immunol (1993) 150:1122–36 7678617

[50] Picker LJ Singh MK Zdraveski Z Treer JR Waldrop SL Bergstresser PR Direct demonstration of cytokine synthesis heterogeneity among human memory/effector T cells by flow cytometry. Blood (1995) 86:1408–19 7632949

[51] Gattinoni L Lugli E Ji Y Pos Z Paulos CM Quigley MF A human memory T cell subset with stem cell-like properties. Nat Med (2011) 17:1290–7 10.1038/nm.2446 21926977 PMC3192229

[52] Lugli E Dominguez MH Gattinoni L Chattopadhyay PK Bolton DL Song K Superior T memory stem cell persistence supports long-lived T cell memory. J Clin Invest (2013) 123:594–9 10.1172/JCI66327 23281401 PMC3561805

[53] Fagnoni FF Vescovini R Passeri G Bologna G Pedrazzoni M Lavagetto G Shortage of circulating naive CD8(^+^) T cells provides new insights on immunodeficiency in aging. Blood (2000) 95:2860–8 10779432

[54] Lugli E Pinti M Nasi M Troiano L Ferraresi R Mussi C Subject classification obtained by cluster analysis and principal component analysis applied to flow cytometric data. Cytometry A (2007) 71:334–44 10.1002/cyto.a.20387 17352421

[55] Fritsch RD Shen X Sims GP Hathcock KS Hodes RJ Lipsky PE Stepwise differentiation of CD4 memory T cells defined by expression of CCR7 and CD27. J Immunol (2005) 175:6489–97 16272303 10.4049/jimmunol.175.10.6489

[56] Okada R Kondo T Matsuki F Takata H Takiguchi M Phenotypic classification of human CD4^+^ T cell subsets and their differentiation. Int Immunol (2008) 20:1189–99 10.1093/intimm/dxn075 18635582

[57] Picker LJ Reed-Inderbitzin EF Hagen SI Edgar JB Hansen SG Legasse A IL-15 induces CD4 effector memory T cell production and tissue emigration in nonhuman primates. J Clin Invest (2006) 116:1514–24 10.1172/JCI27564 16691294 PMC1459071

[58] Lugli E Goldman CK Perera LP Smedley J Pung R Yovandich JL Transient and persistent effects of IL-15 on lymphocyte homeostasis in nonhuman primates. Blood (2010) 116:3238–48 10.1182/blood-2010-03-275438 20631381 PMC2995354

[59] Seder RA Darrah PA Roederer M T-cell quality in memory and protection: implications for vaccine design. Nat Rev Immunol (2008) 8:247–58 10.1038/nri2274 18323851

[60] Bird JJ Brown DR Mullen AC Moskowitz NH Mahowald MA Sider JR Helper T cell differentiation is controlled by the cell cycle. Immunity (1998) 9:229–37 10.1016/S1074-7613(00)80605-6 9729043

[61] Romero P Zippelius A Kurth I Pittet MJ Touvrey C Iancu EM Four functionally distinct populations of human effector-memory CD8^+^ T lymphocytes. J Immunol (2007) 178:4112–9 17371966 10.4049/jimmunol.178.7.4112

[62] Takata H Takiguchi M Three memory subsets of human CD8^+^ T cells differently expressing three cytolytic effector molecules. J Immunol (2006) 177:4330–40 16982867 10.4049/jimmunol.177.7.4330

[63] Chattopadhyay PK Betts MR Price DA Gostick E Horton H Roederer M The cytolytic enzymes granyzme A, granzyme B, and perforin: expression patterns, cell distribution, and their relationship to cell maturity and bright CD57 expression. J Leukoc Biol (2009) 85:88–97 10.1189/jlb.0208107 18820174 PMC2638730

[64] Tomiyama H Takata H Matsuda T Takiguchi M Phenotypic classification of human CD8^+^ T cells reflecting their function: inverse correlation between quantitative expression of CD27 and cytotoxic effector function. Eur J Immunol (2004) 34:999–1010 10.1002/eji.200324478 15048710

[65] Harari A Petitpierre S Vallelian F Pantaleo G Skewed representation of functionally distinct populations of virus-specific CD4 T cells in HIV-1-infected subjects with progressive disease: changes after antiretroviral therapy. Blood (2004) 103:966–72 10.1182/blood-2003-04-1203 12958069

[66] Younes SA Yassine-Diab B Dumont AR Boulassel MR Grossman Z Routy JP HIV-1 Viremia prevents the establishment of interleukin 2-producing HIV-specific memory CD4^+^ T cells endowed with proliferative capacity. J Exp Med (2003) 198:1909–22 10.1084/jem.20031598 14676302 PMC2194146

[67] Semmo N Day CL Ward SM Lucas M Harcourt G Loughry A Preferential loss of IL-2-secreting CD4^+^ T helper cells in chronic HCV infection. Hepatology (2005) 41:1019–28 10.1002/hep.20669 15841456

[68] Zielinski CE Corti D Mele F Pinto D Lanzavecchia A Sallusto F Dissecting the human immunologic memory for pathogens. Immunol Rev (2011) 240:40–51 10.1111/j.1600-065X.2010.01000.x 21349085

[69] Millington KA Innes JA Hackforth S Hinks TS Deeks JJ Dosanjh DP Dynamic relationship between IFN-gamma and IL-2 profile of *Mycobacterium tuberculosis*-specific T cells and antigen load. J Immunol (2007) 178:5217–26 17404305 10.4049/jimmunol.178.8.5217 PMC2743164

[70] Unutmaz D Pileri P Abrignani S Antigen-independent activation of naive and memory resting T cells by a cytokine combination. J Exp Med (1994) 180:1159–64 10.1084/jem.180.3.1159 8064232 PMC2191658

[71] Geginat J Lanzavecchia A Sallusto F Proliferation and differentiation potential of human CD8^+^ memory T-cell subsets in response to antigen or homeostatic cytokines. Blood (2003) 101:4260–6 10.1182/blood-2002-11-3577 12576317

[72] Geginat J Sallusto F Lanzavecchia A Cytokine-driven proliferation and differentiation of human naive, central memory, and effector memory CD4(^+^) T cells. J Exp Med (2001) 194:1711–9 10.1084/jem.194.12.1711 11748273 PMC2193568

[73] Walzl G Ronacher K Hanekom W Scriba TJ Zumla A Immunological biomarkers of tuberculosis. Nat Rev Immunol (2011) 11:343–54 10.1038/nri2960 21475309

[74] Rozot V Vigano S Mazza-Stalder J Idrizi E Day CL Perreau M *Mycobacterium tuberculosis*-specific CD8+ T cells are functionally and phenotypically different between latent infection and active disease. Eur J Immunol (2013) 43:1568–77 10.1002/eji.201243262 23456989 PMC6535091

[75] Marín ND París SC Rojas M García LF Functional profile of CD4+ and CD8+ T cells in latently infected individuals and patients with active TB. Tuberculosis (Edinb) (2013) 93:155–66 10.1016/j.tube.2012.12.002 23332142

[76] Flynn JL Chan J Tuberculosis: latency and reactivation. Infect Immun (2001) 69:4195–201 10.1128/IAI.69.7.4195-4201.2001 11401954 PMC98451

[77] Almeida JR Price DA Papagno L Arkoub ZA Sauce D Bornstein E Superior control of HIV-1 replication by CD8^+^ T cells is reflected by their avidity, polyfunctionality, and clonal turnover. J Exp Med (2007) 204:2473–85 10.1084/jem.20070784 17893201 PMC2118466

[78] Scriba TJ Tameris M Mansoor N Smit E van der Merwe L Isaacs F Modified vaccinia Ankara-expressing Ag85A, a novel tuberculosis vaccine, is safe in adolescents and children, and induces polyfunctional CD4^+^ T cells. Eur J Immunol (2010) 40:279–90 10.1002/eji.200939754 20017188 PMC3044835

[79] Abel B Tameris M Mansoor N Gelderbloem S Hughes J Abrahams D The novel tuberculosis vaccine, AERAS-402, induces robust and polyfunctional CD4^+^ and CD8^+^ T cells in adults. Am J Respir Crit Care Med (2010) 181:1407–17 10.1164/rccm.200910-1484OC 20167847 PMC2894413

[80] Soares AP Scriba TJ Joseph S Harbacheuski R Murray RA Gelderbloem SJ Bacillus Calmette-Guérin vaccination of human newborns induces T cells with complex cytokine and phenotypic profiles. J Immunol (2008) 180:3569–77 18292584 10.4049/jimmunol.180.5.3569 PMC2842001

[81] Beveridge NE Price DA Casazza JP Pathan AA Sander CR Asher TE Immunisation with BCG and recombinant MVA85A induces long-lasting, polyfunctional *Mycobacterium tuberculosis*-specific CD4+ memory T lymphocyte populations. Eur J Immunol (2007) 37:3089–100 10.1002/eji.200737504 17948267 PMC2365909

[82] Petruccioli E Petrone L Vanini V Sampaolesi A Gualano G Girardi E γ/TNFα specific-cells and effector memory phenotype associate with active tuberculosis. J Infect (2013) 66:475–86 10.1016/j.jinf.2013.02.004 23462597

[83] Ciuffreda D Comte D Cavassini M Giostra E Bühler L Perruchoud M Polyfunctional HCV-specific T-cell responses are associated with effective control of HCV replication. Eur J Immunol (2008) 38:2665–77 10.1002/eji.200838336 18958874

[84] Chiacchio T Petruccioli E Vanini V Butera O Cuzzi G Petrone L Higher frequency of T-cell response to *M. tuberculosis* latency antigen Rv2628 at the site of active tuberculosis disease than in peripheral blood. PLoS One (2011) 6:e27539 10.1371/journal.pone.0027539 22102905 PMC3213161

[85] El Fenniri L Toossi Z Aung H El Iraki G Bourkkadi J Benamor J Polyfunctional *Mycobacterium tuberculosis*-specific effector memory CD4^+^ T cells at sites of pleural TB. Tuberculosis (Edinb) (2011) 91:224–30 10.1016/j.tube.2010.12.005 21459675 PMC3306579

[86] Sargentini V Mariotti S Carrara S Gagliardi MC Teloni R Goletti D Cytometric detection of antigen-specific IFN-gamma/IL-2 secreting cells in the diagnosis of tuberculosis. BMC Infect Dis (2009) 9:99 10.1186/1471-2334-9-99 19549330 PMC2708166

[87] Caccamo N Guggino G Meraviglia S Gelsomino G Di Carlo P Titone L Analysis of *Mycobacterium tuberculosis*-specific CD8 T-cells in patients with active tuberculosis and in individuals with latent infection. PLoS One (2009) 4:e5528 10.1371/journal.pone.0005528 19436760 PMC2678250

[88] Sester U Fousse M Dirks J Mack U Prasse A Singh M Whole-blood flow-cytometric analysis of antigen-specific CD4 T-cell cytokine profiles distinguishes active tuberculosis from non-active states. PLoS One (2011) 6:e17813 10.1371/journal.pone.0017813 21423578 PMC3058054

[89] Young JM Adetifa IM Ota MO Sutherland JS Expanded polyfunctional T cell response to mycobacterial antigens in TB disease and contraction post-treatment. PLoS One (2010) 5:e11237 10.1371/journal.pone.0011237 20574540 PMC2888639

[90] Sutherland JS Adetifa IM Hill PC Adegbola RA Ota MO Pattern and diversity of cytokine production differentiates between *Mycobacterium tuberculosis* infection and disease. Eur J Immunol (2009) 39:723–9 10.1002/eji.200838693 19224636

[91] Caccamo N Guggino G Joosten SA Gelsomino G Di Carlo P Titone L Multifunctional CD4(^+^) T cells correlate with active *Mycobacterium tuberculosis* infection. Eur J Immunol (2010) 40:2211–20 10.1002/eji.201040455 20540114

[92] Harari A Rozot V Enders FB Perreau M Stalder JM Nicod LP Dominant TNF-α^+^ *Mycobacterium tuberculosis*-specific CD4^+^ T cell responses discriminate between latent infection and active disease. Nat Med (2011) 17:372–6 10.1038/nm.2299 21336285 PMC6570988

[93] Day CL Abrahams DA Lerumo L Janse van Rensburg E Stone L O’Rie T Functional capacity of *Mycobacterium tuberculosis*-specific T cell responses in humans is associated with mycobacterial load. J Immunol (2011) 187:2222–32 10.4049/jimmunol.1101122 21775682 PMC3159795

[94] Mueller H Detjen AK Schuck SD Gutschmidt A Wahn U Magdorf K *Mycobacterium tuberculosis*-specific CD4^+^, IFNgamma^+^, and TNFalpha^+^ multifunctional memory T cells coexpress GM-CSF. Cytokine (2008) 43:143–8 10.1016/j.cyto.2008.05.002 18603443

[95] Casey R Blumenkrantz D Millington K Montamat-Sicotte D Kon OM Wickremasinghe M Enumeration of functional T-cell subsets by fluorescence-immunospot defines signatures of pathogen burden in tuberculosis. PLoS One (2010) 5:e15619 10.1371/journal.pone.0015619 21179481 PMC3001879

[96] Wang X Cao Z Jiang J Niu H Dong M Tong A Association of mycobacterial antigen-specific CD4(^+^) memory T cell subsets with outcome of pulmonary tuberculosis. J Infect (2010) 60:133–9 10.1016/j.jinf.2009.10.048 19878691

[97] Schuetz A Haule A Reither K Ngwenyama N Rachow A Meyerhans A Monitoring CD27 expression to evaluate *Mycobacterium tuberculosis* activity in HIV-1 infected individuals in vivo. PLoS One (2011) 6:e27284 10.1371/journal.pone.0027284 22087280 PMC3210152

[98] Pollock KM Whitworth HS Montamat-Sicotte DJ Grass L Cooke GS Kapembwa MS T-cell immunophenotyping distinguishes active from latent tuberculosis. J Infect Dis (2013) 208:952–68 10.1093/infdis/jit265 23966657 PMC3749005

[99] Lalvani A Brookes R Wilkinson RJ Malin AS Pathan AA Andersen P Human cytolytic and interferon gamma-secreting CD8^+^ T lymphocytes specific for *Mycobacterium tuberculosis*. Proc Natl Acad Sci U S A (1998) 95:270–5 10.1073/pnas.95.1.270 9419365 PMC18198

[100] Ladel CH Daugelat S Kaufmann SH Immune response to *Mycobacterium bovis* bacille Calmette Guérin infection in major histocompatibility complex class I- and II-deficient knock-out mice: contribution of CD4 and CD8 T cells to acquired resistance. Eur J Immunol (1995) 25:377–84 10.1002/eji.1830250211 7875199

[101] Ottenhoff TH Lewinsohn DA Lewinsohn DM Human CD4 and CD8 T cell responses to *Mycobacterium tuberculosis*: antigen specificity, function, implications and applications. In: Handbook of Tuberculosis: Immunology and Cell Biology. Weinheim: Wiley-VCH (2008). p. 119–56

[102] Brighenti S Andersson J Induction and regulation of CD8^+^ cytolytic T cells in human tuberculosis and HIV infection. Biochem Biophys Res Commun (2010) 396:50–7 10.1016/j.bbrc.2010.02.141 20494110

[103] Chen CY Huang D Wang RC Shen L Zeng G Yao S A critical role for CD8 T cells in a nonhuman primate model of tuberculosis. PLoS Pathog (2009) 5:e1000392 10.1371/journal.ppat.1000392 19381260 PMC2663842

[104] Mazzaccaro RJ Stenger S Rock KL Porcelli SA Brenner MB Modlin RL Cytotoxic T lymphocytes in resistance to tuberculosis. Adv Exp Med Biol (1998) 452:85–101 10.1007/978-1-4615-5355-7_11 9889963

[105] Levine B Mizushima N Virgin HW Autophagy in immunity and inflammation. Nature (2011) 469:323–35 10.1038/nature09782 21248839 PMC3131688

[106] Deretic V Autophagy in infection. Curr Opin Cell Biol (2010) 22:252–62 10.1016/j.ceb.2009.12.009 20116986 PMC2866841

[107] Joosten SA van Meijgaarden KE van Weeren PC Kazi F Geluk A Savage ND *Mycobacterium tuberculosis* peptides presented by HLA-E molecules are targets for human CD8 T-cells with cytotoxic as well as regulatory activity. PLoS Pathog (2010) 6:e1000782 10.1371/journal.ppat.1000782 20195504 PMC2829052

[108] Heinzel AS Grotzke JE Lines RA Lewinsohn DA McNabb AL Streblow DN HLA-E-dependent presentation of Mtb-derived antigen to human CD8^+^ T cells. J Exp Med (2002) 196:1473–81 10.1084/jem.20020609 12461082 PMC2194265

[109] Cohen NR Garg S Brenner MB Antigen presentation by CD1 lipids, T cells, and NKT cells in microbial immunity. Adv Immunol (2009) 102:1–94 10.1016/S0065-2776(09)01201-2 19477319

[110] Bruns H Meinken C Schauenberg P Härter G Kern P Modlin RL Anti-TNF immunotherapy reduces CD8^+^ T cell-mediated antimicrobial activity against *Mycobacterium tuberculosis* in humans. J Clin Invest (2009) 119:1167–77 10.1172/JCI38482 19381021 PMC2673881

[111] Stenger S Mazzaccaro RJ Uyemura K Cho S Barnes PF Rosat JP Differential effects of cytolytic T cell subsets on intracellular infection. Science (1997) 276:1684–7 10.1126/science.276.5319.1684 9180075

[112] Semple PL Watkins M Davids V Krensky AM Hanekom WA Kaplan G Induction of granulysin and perforin cytolytic mediator expression in 10-week-old infants vaccinated with BCG at birth. Clin Dev Immunol (2011) 2011:438463 10.1155/2011/438463 21234358 PMC3018618

[113] Rahman S Gudetta B Fink J Granath A Ashenafi S Aseffa A Compartmentalization of immune responses in human tuberculosis: few CD8^+^ effector T cells but elevated levels of FoxP3^+^ regulatory t cells in the granulomatous lesions. Am J Pathol (2009) 174:2211–24 10.2353/ajpath.2009.080941 19435796 PMC2684186

[114] Andersson J Samarina A Fink J Rahman S Grundström S Impaired expression of perforin and granulysin in CD8^+^ T cells at the site of infection in human chronic pulmonary tuberculosis. Infect Immun (2007) 75:5210–22 10.1128/IAI.00624-07 17664265 PMC2168267

[115] Kagina BM Abel B Scriba TJ Hughes EJ Keyser A Soares A Specific T cell frequency and cytokine expression profile do not correlate with protection against tuberculosis after Bacillus Calmette-Guérin vaccination of newborns. Am J Respir Crit Care Med (2010) 182:1073–9 10.1164/rccm.201003-0334OC 20558627 PMC2970848

[116] Smith SG Lalor MK Gorak-Stolinska P Blitz R Beveridge NE Worth A *Mycobacterium tuberculosis* PPD-induced immune biomarkers measurable in vitro following BCG vaccination of UK adolescents by multiplex bead array and intracellular cytokine staining. BMC Immunol (2010) 11:35 10.1186/1471-2172-11-35 20609237 PMC2910033

[117] Caccamo N Meraviglia S La Mendola C Guggino G Dieli F Salerno A Phenotypical and functional analysis of memory and effector human CD8 T cells specific for mycobacterial antigens. J Immunol (2006) 177:1780–5 16849488 10.4049/jimmunol.177.3.1780

[118] Leyten EM Lin MY Franken KL Friggen AH Prins C van Meijgaarden KE Human T-cell responses to 25 novel antigens encoded by genes of the dormancy regulon of *Mycobacterium tuberculosis*. Microbes Infect (2006) 8:2052–60 10.1016/j.micinf.2006.03.018 16931093

[119] Roupie V Romano M Zhang L Korf H Lin MY Franken KL Immunogenicity of eight dormancy regulon-encoded proteins of *Mycobacterium tuberculosis* in DNA-vaccinated and tuberculosis-infected mice. Infect Immun (2007) 75:941–9 10.1128/IAI.01137-06 17145953 PMC1828490

[120] Schuck SD Mueller H Kunitz F Neher A Hoffmann H Franken KL Identification of T-cell antigens specific for latent *mycobacterium tuberculosis* infection. PLoS One (2009) 4:e5590 10.1371/journal.pone.0005590 19440342 PMC2680040

[121] Flynn JL Goldstein MM Triebold KJ Koller B Bloom BR Major histocompatibility complex class I-restricted T cells are required for resistance to *Mycobacterium tuberculosis* infection. Proc Natl Acad Sci U S A (1992) 89:12013–7 10.1073/pnas.89.24.12013 1465432 PMC50688

[122] Commandeur S Lin MY van Meijgaarden KE Friggen AH Franken KL Drijfhout JW Double-and monofunctional CD4ss^+^ and CD8^+^ T-cell responses to *Mycobacterium tuberculosis* DosR antigens and peptides in long-term latently infected individuals. Eur J Immunol (2011) 41:2925–36 10.1002/eji.201141602 21728172

[123] Nyendak MR Park B Null MD Baseke J Swarbrick G Mayanja-Kizza H Tuberculosis research unit and the tuberculosis trials consortium. *Mycobacterium tuberculosis* specific CD8(^+^) T cells rapidly decline with antituberculosis treatment. PLoS One (2013) 8:e81564 10.1371/journal.pone.0081564 24324704 PMC3852504

[124] Kunnath-Velayudhan S Davidow AL Wang HY Molina DM Huynh VT Salamon H Proteome-scale antibody responses and outcome of *Mycobacterium tuberculosis* infection in nonhuman primates and in tuberculosis patients. J Infect Dis (2012) 206:697–705 10.1093/infdis/jis421 22732925 PMC3491745

[125] Harari A Dutoit V Cellerai C Bart PA Du Pasquier RA Pantaleo G Functional signatures of protective antiviral T-cell immunity in human virus infections. Immunol Rev (2006) 211:236–54 10.1111/j.0105-2896.2006.00395.x 16824132

[126] Lewinsohn DA Heinzel AS Gardner JM Zhu L Alderson MR Lewinsohn DM *Mycobacterium tuberculosis*-specific CD8^+^ T cells preferentially recognize heavily infected cells. Am J Respir Crit Care Med (2003) 168:1346–52 10.1164/rccm.200306-837OC 12969871

[127] Lancioni C Nyendak M Kiguli S Zalwango S Mori T Mayanja-Kizza H Tuberculosis research unit. CD8^+^ T cells provide an immunologic signature of tuberculosis in young children. Am J Respir Crit Care Med (2012) 185:206–12 10.1164/rccm.201107-1355OC 22071329 PMC3297089

[128] Flynn Diedrich CR Flynn JL HIV-1/*Mycobacterium tuberculosis* coinfection immunology: how does HIV-1 exacerbate tuberculosis? Infect Immun (2011) 79:1407–17 10.1128/IAI.01126-10 21245275 PMC3067569

[129] Nambuya A Sewankambo N Mugerwa J Goodgame R Lucas S *Tuberculous lymphadenitis* associated with human immunodeficiency virus (HIV) in Uganda. J Clin Pathol (1988) 41:93–6 10.1136/jcp.41.1.93 3343383 PMC1141342

[130] Pitchenik AE Burr J Suarez M Fertel D Gonzalez G Moas C Human T-cell lymphotropic virus-III (HTLV-III) seropositivity and related disease among 71 consecutive patients in whom tuberculosis was diagnosed. A prospective study. Am Rev Respir Dis (1987) 135:875–9 3645999 10.1164/arrd.1987.135.4.875

[131] Selwyn PA Sckell BM Alcabes P Friedland GH Klein RS Schoenbaum EE High risk of active tuberculosis in HIV-infected drug users with cutaneous anergy. JAMA (1992) 268:504–9 10.1001/jama.1992.03490040080029 1619742

[132] Lawn SD Butera ST Shinnick TM Tuberculosis unleashed: the impact of human immunodeficiency virus infection on the host granulomatous response to *Mycobacterium tuberculosis*. Microbes Infect (2002) 4:635–46 10.1016/S1286-4579(02)01582-4 12048033

[133] WHO Publications, Guidelines, Reports on HIV/AIDS Available from: http://www.who.int/publications/en/

[134] Geldmacher C Zumla A Hoelscher M Interaction between HIV and *Mycobacterium tuberculosis*: HIV-1-induced CD4 T-cell depletion and the development of active tuberculosis. Curr Opin HIV AIDS (2012) 7:268–75 10.1097/COH.0b013e3283524e32 22495739

[135] Sonnenberg P Glynn JR Fielding K Murray J Godfrey-Faussett P Shearer S How soon after infection with HIV does the risk of tuberculosis start to increase? A retrospective cohort study in South African gold miners. J Infect Dis (2005) 191:150–8 10.1086/426827 15609223

[136] Sonnenberg P Murray J Glynn JR Shearer S Kambashi B Godfrey-Faussett P HIV-1 and recurrence, relapse, and reinfection of tuberculosis after cure: a cohort study in South African mineworkers. Lancet (2001) 358:1687–93 10.1016/S0140-6736(01)06712-5 11728545

[137] Lawn SD Myer L Edwards D Bekker LG Wood R Short-term and long-term risk of tuberculosis associated with CD4 cell recovery during antiretroviral therapy in South Africa. AIDS (2009) 23:1717–25 10.1097/QAD.0b013e32832d3b6d 19461502 PMC3801095

[138] Kizza HM Rodriguez B Quinones-Mateu M Mirza M Aung H Yen-Lieberman B Persistent replication of human immunodeficiency virus type 1 despite treatment of pulmonary tuberculosis in dually infected subjects. Clin Diagn Lab Immunol (2005) 12:1298–304 16275944 10.1128/CDLI.12.11.1298-1304.2005 PMC1287765

[139] Geldmacher C Schuetz A Ngwenyama N Casazza JP Sanga E Saathoff E Early depletion of *Mycobacterium tuberculosis* specific T helper 1 cell responses after HIV-1 infection. J Infect Dis (2008) 198:1590–8 10.1086/593017 19000013 PMC2650495

[140] Kalsdorf B Scriba TJ Wood K Day CL Dheda K Dawson R HIV-1 infection impairs the bronchoalveolar T-cell response to mycobacteria. Am J Respir Crit Care Med (2009) 180:1262–70 10.1164/rccm.200907-1011OC 19797156 PMC2796736

[141] Mansoor N Scriba TJ de Kock M Tameris M Abel B Keyser A HIV-1 infection in infants severely impairs the immune response induced by Bacille Calmette–Guerin vaccine. J Infect Dis (2009) 199:982–90 10.1086/597304 19236280 PMC2815505

[142] Rangaka MX Diwakar L Seldon R van Cutsem G Meintjes GA Morroni C Clinical, immunological, and epidemiological importance of antituberculosis T cell responses in HIV-infected Africans. Clin Infect Dis (2007) 44:1639–46 10.1086/518234 17516410

[143] Matthews K Ntsekhe M Syed F Scriba T Russell J Tibazarwa K HIV-1 infection alters CD4+ memory T-cell phenotype at the site of disease in extrapulmonary tuberculosis. Eur J Immunol (2012) 42:147–57 10.1002/eji.201141927 22215422 PMC3298896

[144] Wilkinson KA Seldon R Meintjes G Rangaka MX Hanekom WA Maartens G Dissection of regenerating T-cell responses against tuberculosis in HIV-infected adults sensitized by *Mycobacterium tuberculosis*. Am J Respir Crit Care Med (2009) 180:674–83 10.1164/rccm.200904-0568OC 19628776 PMC4176738

[145] Day CL Mkhwanazi N Reddy S Mncube Z van der Stok M Klenerman P Detection of polyfunctional *Mycobacterium tuberculosis*-specific T cells and association with viral load in HIV-1-infected persons. J Infect Dis (2008) 197:990–9 10.1086/529048 18419535 PMC5688849

[146] Weiner J Maertzdorf J Kaufmann SH The dual role of biomarkers for understanding basic principles and devising novel intervention strategies in tuberculosis. Ann N Y Acad Sci (2013) 1283:22–9 10.1111/j.1749-6632.2012.06802.x 23181737

